# Longitudinal Effects of Moderate to Vigorous Physical Activity in Physical Education Classes on Attention and Academic Achievement

**DOI:** 10.3390/bs14110982

**Published:** 2024-10-22

**Authors:** Kyulee Shin, Sukkyung You, Mihye Kim

**Affiliations:** 1 Department of Sports Sciences, Seoul National University of Science & Technology, 232 Gongneung-ro, Nowon-gu, Seoul 01811, Republic of Korea; kyuleeshin@seoultech.ac.kr; 2 College of Education, Hankuk University of Foreign Studies, 107 Imun-dong, Dongdaemun-gu, Seoul 02450, Republic of Korea; 3 Korea Institute of Sport Science, Olympic Cultural Center, 424 Olympic-ro, Songpa-gu, Seoul 05540, Republic of Korea

**Keywords:** moderate to vigorous physical activity, physical education classes, academic achievement, attention, KCYPS2010

## Abstract

Previous studies showed moderate to vigorous physical activity (MVPA) and aerobic fitness in adolescents are significant factors for cognitive and academic performance. Most previous studies have employed a cross-sectional design; consequently, the evidence on the longitudinal effect of physical education classes (PECs) on cognitive performance and academic achievement is limited. Therefore, the current study utilized a longitudinal design to examine the longstanding effect of MVPA during PECs on cognitive and academic performance across gender groups. Structural equation modeling analyses were employed to understand how MVPA influences youth academic achievement in a nationally representative sample (*n* = 2092). Study findings indicated that (a) MVPA exerted a direct effect on initial academic achievement as well as an indirect effect, which is mediated by middle school students’ attention in both gender groups; (b) MVPA had both long-term direct effects on academic achievement as well as indirect effects on attention, which ultimately affected the subsequent academic achievements of female middle school students.

## 1. Introduction

Cognition is the psychological activity of gaining knowledge and using it [1]. The main cognitive processes consist of attention, memory, learning, reasoning, decision making, problem-solving, and creativity [2]. Attention is classified as focusing on one thing while ignoring other stimuli and distractions [3]. Attention plays a central role in self-regulation [4] and memory [5] for children and adolescents and contributes academic development [6].

In the distant past, most cognitive scientists viewed cognition as a product of the brain. However, since the 1990s, a new perspective has emerged that cognitive is not only produced in the brain but also through bodily interaction with its environment [7]. The embodied cognition theory (ECT) has been adopted as a theoretical explanatory model of how the human body and brain work together in neuroscience and psychology [8]. According to the ECT, physical and visual bodily stimuli, as well as auditory tasks, can improve attention and academic achievement [9,10,11].

Recently, sports scientists have been actively discussing ECT, and it has emerged as a hot topic among sports researchers [12,13]. Empirical evidence now exists that physical activity (PA) has a positive effect on attention. For example, moderate-to-vigorous physical activity (MVPA) improved tasked-related attention in adolescents [14]. During periods of MVPA, neurotransmitters such as dopamine and norepinephrine are released [15,16]. These chemicals play important roles in improving attention [17]. Furthermore, MVPA has shown to improve academic achievement such as reading, mathematics, and science in children and adolescents [18,19,20,21].

Physical education classes (PECs) offer the inducement of MVPA and can therefore be a critical means to the above [22,23,24]. It is of interest that academic achievement was significantly associated with MVPA in PECs [25,26,27], although not with PECs alone [22,25], as MVPA must be included.

For Korean middle school students, the primary participants of this study, PECs offer a universal opportunity for participation in MVPA. Middle schools provide after-school sports activities, but there is a high probability that they will not be selected because they are no longer mandatory [28]. The college admittance process starts to take precedence at this age in Korean society, and Korean parents do not enroll their children in non-scholastic extra-curricular activities [29].

Gender, age, and puberty status are important factors related to adolescents’ physical, cognitive, and academic performance [30]. Gender, of the above, impacts PA participation the most [31]. Male students are more involved and perform better in sports and exercise than their female counterparts. Considering boys and girls have different psychological values over physical activity and their relative importance varies, there are gender differences that exist in the influence of physical activity on cognitive performance [32].

In short, previous studies concluded a positive association between MVPA in PECs and attention [14,17] and between MVPA in PECs and academic performance [18,19,20,21]. However, they lacked larger sample sizes that took gender differences into account. Furthermore, most previous studies employed a cross-sectional design [11,20,21]; thus, the evidence on the long-term effects of MVPA in PECs on attention and academic performance is limited [30].

Therefore, based on the embodied cognition model, the current study utilized a longitudinal design to examine the longitudinal effect of MVPA in PECs on cognitive and academic performance across gender groups. The aim of this study was to identify the long-term impact of adolescents’ PA on academic achievement using latent growth modeling. Specifically, we examined the influence of MVPA in PECs on academic achievement in reading, mathematics, and science from the 7th grade through to the 9th grade in Korean middle schools.

## 2. Methods

### 2.1. Participants and Procedure

We utilized a longitudinal sample of 2092 middle school students from the Korean Children and Youth Panel Survey (KCYPS) data. The KCYPS is a six-year longitudinal study conducted by the National Youth Policy Institute, funded by the Korean national government. The survey design includes a clustered, stratified national probability sample from approximately 85 elementary schools. This survey was initially completed in 2010 when all students were in their fourth year of elementary school; their mean age was ten. Students then re-took the survey every last quarter of the year until 2016. The current study utilized secondary data; therefore, ethical approval was not required.

The current study samples are drawn from the last three years of the panel data (i.e., 2013 to 2015) when the students were in the 7th to 9th grades. The data used for study analyses comprised a longitudinal panel of 2092 participants who were the same students evaluated three times across the 7th to 9th grades. This sample included 992 (47.4%) girls and 1100 (52.5%) boys, whose ages ranged from 13 to 15 years (M = 14.01; SD = 0.82).

### 2.2. Measures

#### 2.2.1. Attention

We used the Korean Adolescent Behavior Checklist (K-ABCL) to measure students’ attention levels [33]. We used seven items from K-ABCL to measure attention (e.g., When I need to concentrate and solve a problem, I have trouble focusing my attention). All items were coded so that higher scores indicated a higher level of attention during the class. Cronbach’s alpha coefficient for this measure was 0.79 in this sample.

Several studies used this scale with South Korean adolescents [34,35,36,37]. The results of the studies showed that adolescents’ emotional and behavioral problems were significantly related to their attention. For instance, attention was found to be related to study habits and future goals [34], school adjustment [37], and depression [37]. Furthermore, adolescents’ self-control and attention were found to impact cell phone dependency significantly [36]. The Cronbach alphas in these studies ranged from 0.79 to 0.84, indicating satisfactory reliability. These findings support the validity of the instrument for use with this population.

#### 2.2.2. Academic Achievement

We used the nationwide achievement test, i.e., the Korean Scholastic Achievement Test (KSAT). This test was designed for the accurate measurement of the ability of individuals at a given time point and of their achievement growth over time. This test was vertically equated with offering a measurement of gain across time. The outcome variable measuring academic achievement was constructed on three assessment scores: the item response theory (IRT) theta scores of readings, math, and science when students were 7th graders (i.e., in their first year in middle school).

#### 2.2.3. Physical Activity

We used students’ self-evaluations of MVPA during a week of PECs. The students were asked to compare exercise time to the extent of sweating during PECs. Students answered on a five-point Likert scale for this item (0 = not at all, 4 = 4 h or more). The higher the score, the longer the time spent engaging in MVPA.

### 2.3. Statistical Analysis

**Figure 1:**
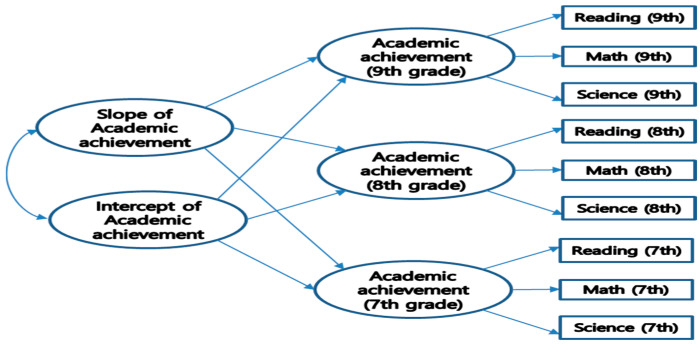
Unconditional latent growth model of academic achievement.

Study analyses were conducted in three stages. In stage one, we conducted t-tests and intercorrelations among variables. In the second stage, we conducted a univariate linear latent growth model (LGM) to test the measurement model for the three subjects. In the third stage, we conducted structural equation modeling (SEM) analysis (see Figure 1) to test the hypothesized models.

#### Model Evaluation

Model fit was decided based on several criteria: comparative fit index (CFI; [38]), root mean square error of approximation (RMSEA; [39]), and non-normed fit index (NNFI; [40]). Values smaller than 0.08 for the RMSEA and close to 0.95 for the NNFI and CFI were used to decide on a better fitting model. Analyses were performed using Mplus [41].

We used the nonparametric bootstrapping method [42,43] to assess the significance of mediating effects. This approach repeatedly employs sampling from the given data and assesses the indirect influence in each resampled dataset.

## 3. Results

### 3.1. Descriptive Statistics

We used *t*-tests to examine gender differences (see Table 1). Male students participated longer in PA than females, and female students showed a higher level of attention than male students. The findings showed that adolescents’ PA, attention, and academic achievement scores were significantly correlated for both gender groups.

### 3.2. Stage I: Latent Growth Model Analysis

We conducted a univariate linear LGM (see path diagram in Figure 1) to see if academic achievement was stable or changed during adolescence. The intercept factor loadings were set to 1.0, representing the initial starting point of the growth curve at Time 1. The slope factor loadings were set to 0, 1, and 2 for three time points representing the linear growth function. The univariate linear models were acceptable based on fit indices. The value of CFI was 0.981, and the value of RMSEA was 0.066.

### 3.3. Stage II: Testing the Hypothesized Models

**Figure 2:**
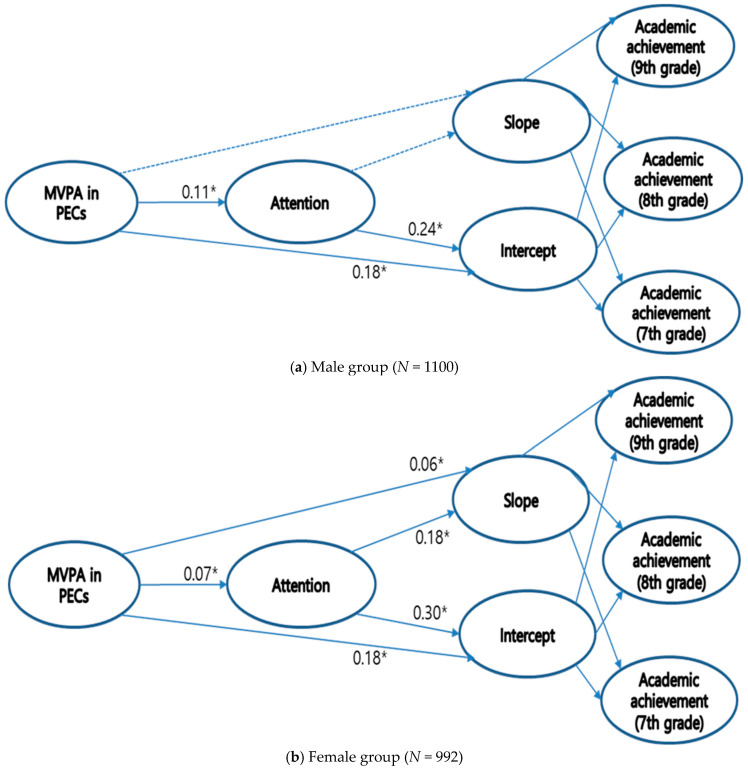
Final model estimation with standardized beta coefficients (*N* = 2092) for the (**a**) male group and (**b**) female group. Note. * *p* < 0.05; Significant path coefficients are shown with a bold line; dotted line = non-significant.

We examined two models. The initial structural model (model 1) was a model with direct and indirect paths from PA to academic achievement through a mediating variable. The second structural model (model 2) was a model without a direct path from PA to academic achievement. For boys, model 1 produced an overall χ^2^ (107) value of 664.69, with CFI = 0.951, NNFI = 0.955, and RMSEA = 0.065, and model 2 produced an overall χ^2^(108) value of 702.84, with CFI = 0.937, NNFI = 0.910, and RMSEA = 0.071. For girls, model 1 produced an overall χ^2^ (107) value of 540.04, with CFI = 0.953, NNFI = 0.954, and RMSEA = 0.064, and model 2 yielded an overall χ^2^(108) value of 595.41, with CFI = 0.938, NNFI = 0.913, and RMSEA = 0.070. A chi-square difference test endorsed model 2. Thus, we selected model 2 as the final theoretical model (see Figure 2). The results showed that there were gender differences in the two paths. Specifically, for the path from MVPA and attention to slope of academic achievement, the effects were significant for girls only (*β* = 0.06 and *β* = 0.18, *p* < 0.05, respectively). Paths from MVPA to attention and MVPA and attention to intercepts of academic achievement were significant for both gender groups. Standardized beta coefficients for these paths are shown in Figure 2.

Concerning the mediating effects, the bootstrap test outcomes specify that the indirect effects of PA on the intercept of academic achievement through attention (*β* = 0.03, and *β* = 0.02, *p* < 0.05, for girls and boys, correspondingly) and on the slope of academic achievement for girls (*β* = 0.02, *p* < 0.05) were significant.

## 4. Discussion

This study sought to identify the long-term impact of adolescents’ PA on academic achievement using latent growth modeling. Specifically, we examined the influence of MVPA in PECs on academic achievement in reading, math, and science from the 7th grade through to the 9th grade in Korean middle schools.

First, the findings of the current study showed that MVPA in PECs directly and indirectly affects academic achievement by mediating variable attention for both gender groups. Such outcomes can be explained by the embodied cognition theory (ECT) [8], which emphasizes the connection between better fitness and higher cognitive performance. These findings align with previous findings in that PECs did not have detrimental effects on academic achievement; instead, it exerted a favorable effect on achievement in classroom settings [44,45]. Physical activity aids in excluding all other thoughts and emotions by immersing them in a particular activity [46]. This is frequently mentioned in studies on flow experience in sports [46]. Mainly, in exercise, a clear goal for a task is posted, and a sports instructor provides an individual with a challenging task of difficulty that can only be performed beyond the person’s average capacity to perform the task. In such a situation, the individual cannot focus on any external distracting thoughts but concentrates solely on the task by organizing and analyzing the collected information [3]. Therefore, exercise provides a sense of control over the activity and encourages pursuing the activity again by rewarding pleasure [47]. The results imply the value of PE since PA positively affects adolescents’ cognitive development, which may improve their academic achievement [48].

Second, study findings showed that there are gender differences. For boys, MVPA in PECs has exerted direct and indirect effects only on initial academic achievement (7th grade) through attention. These results corroborate previous research. The long-term effect of PA on academic achievement was confirmed significantly for girls only, not for boys [44,49]. The gender difference in our findings is in accordance with previous research in that girls experience more significant effects of physical fitness on academic achievement measures than boys. Since boys are generally more physically robust than girls, the stimulus gained during co-ed PE classes may not be satisfactory for boys. Therefore, there is a need to offer the optimal level of PA for boys, which may result in improved cognitive and academic performance in boys.

Third, for girls, study findings showed that MVPA in PECs had both direct and indirect effects on initial academic achievement (7th grade) through attention and on the slope of academic achievement (7th through 9th grade). These findings align with previous studies in that the greater intensity of PA leads to higher academic achievement for girls and not for boys [50,51]. The difference in our findings between genders may be explained by physiological and psychological mechanisms. Adolescent girls have lower fitness levels [52,53] and lower self-esteem than adolescent boys [54,55]. Therefore, these variables can cause differences in the relationship between PA and academic achievement. This might explain that MVPA in PEC can positively influence educational outcomes and psychological domains, and these effects may vary across gender groups. Extant research suggests that psychological factors (i.e., self-esteem and depression) are associated with PA and academic achievement [56,57]. Therefore, these potential confounding variables should be considered in future studies so that gender-specific prevention programming can be implemented in school settings.

## 5. Limitations

This study has several limitations. First, this study utilized a secondary dataset. Because the PE class information could not be obtained, it is impossible to know which type of class content and which teaching methods effectively increased attention and academic achievement. More research is needed to consider the content of PE. Second, this study relied on self-reported measures. Future research is needed to include more various measures, including observational data. Third, students’ PA was measured based on self-reporting. Future researchers should record students’ PA using more objective measures (e.g., accelerometers, pedometers). Fourth, adolescents’ PA can vary depending on their physiological background and possible confounding variables (e.g., self-esteem, depression, perceived puberty status). Therefore, future research needs to address a more comprehensive understanding of PE and cognitive and academic development across gender groups.

## 6. Conclusions

In conclusion, the current study findings have filled the gaps in extant research that contribute to our understanding of the mechanisms of MVPA on academic achievement by investigating adolescents’ attention as an internal mediating factor across gender groups. The current study findings are valuable in that the results are based on nationally representative longitudinal panel data along with the utilization of advanced statistical methods. Empirical studies examining the longitudinal relationship among PA, attention, and academic achievement using a non-Western sample are very scarce. Thus, our study is significant because it has prepared primary data for follow-up studies by verifying the relationship among these variables for adolescents.

In this study, we found that more time spent in MVPA helped adolescents function better cognitively and perform better academically. Outcomes were more favorable for girls. The outcome of the current study confirmed the argument proposed by the embodied cognition theory that improved attention through PA leads to enhanced academic achievement. It emphasizes the need to develop distinct support for a viable brain/body educational intervention based on the embodied cognition model. Previous studies report that a motivational environment constructed by a PE teacher, that is, task-oriented and mastery-oriented rather than performance-oriented, plays a vital role in students’ cognitive activity [58]. In addition, PE teachers are reported to promote cognitive activity in PE classes by supporting students to create their performance routines and participate in self-directed classes rather than dedicating class time to teaching students perfect forms [59]. This study could not confirm teachers’ class curricula because panel data were used. However, it can be suggested that the PE teachers’ class contents are a significant factor in cognitive development [60,61]. Schools should offer PE programs for their many beneficial influences on students’ cognitive and academic development. Furthermore, school administrators and teachers strive to provide gender-sensitive PE programs that reflect gender differences in physical fitness. Therefore, promoting MVPA can be a crucial educational intervention strategy in school settings.

## Figures and Tables

**Table 1:** Correlation and descriptive statistics for study variables by gender.

	1	2	3
1. MVPA in PEC ^α^	-	0.07 *	0.11 *
2. Attention ^α^	0.10 *	-	0.30 *
3. Academic achievement ^α^ Means (SD)	0.14 *	0.20 *	-
Male	3.68 (1.17)	2.77 (0.54)	4.58 (2.36)
Female	2.92 (1.33)	2.82 (0.52)	3.99 (2.12)

Note. Correlations for female students are diagonal; ^α^ Gender difference is significant at * *p* < 0.05.

## Data Availability

Data are available from NYPI Youth and Children Data Archive form https://www.nypi.re.kr/archive/board?menuId=MENU00329 (accessed on 13 January 2024) with the permission of National Youth Policy Institute.
